# Meeting on Establishment of Consortium to Study Invasive Salmonelloses in Sub-Saharan Africa

**DOI:** 10.3201/eid1507.090416

**Published:** 2009-07

**Authors:** John D. Clemens

**Affiliations:** Author affiliation: International Vaccine Institute, Seoul, Republic of Korea

Estimates of typhoid fever in Africa have been based exclusively on placebo group incidence rates during vaccine trials in the Arab Republic of Egypt and the Republic of South Africa (*1**–**4*); population-based data on infections caused by *Salmonella enterica* serotype Typhi have not been available. In the Gambia, Mabey et al. described a series of 5,466 febrile patients hospitalized during 1979–1984 from whom blood cultures were taken (*5*). Of the 246 blood cultures from which bacteria were isolated, 45 (18%) were positive for *Salmonella* serotype Typhi and 71 (28%) were positive for non-Typhi serotypes of *S. enterica*. The importance of *S. enterica* non-Typhi serotypes in febrile illness also has been demonstrated in other studies (*6**–**9*), including a population-based study (*9*). In that study, of 1,094 children with bacteremia admitted to the hospital during 1998–2002 in Kilifi, Kenya, 166 (15%) had *S. enterica* non-Typhi serotypes and 1 had *Salmonella* Typhi; *S. enterica* non-Typhi serotypes were the second most frequently isolated bacterial species.

The few published data do not clarify whether *Salmonella* Typhi causes as much disease and death in sub-Saharan Africa as in Asia. This uncertainty is an impediment to public health decisions about the deployment of modern typhoid vaccines, which are inexpensive, safe, and effective. Bacteremia from other salmonellae are increasingly recognized to cause considerable illness in sub-Saharan Africa; this finding contrasts with reports from industrialized countries, where *S. enterica* non-Typhi serotypes are associated mainly with noninvasive illness, such as gastroenteritis (*10*). The relative importance of invasive *Salmonella* infections in sub-Saharan Africa is likely to increase as the other major causes of bacteremia, *Streptococcus pneumoniae* and *Haemophilus influenzae* type b (Hib), are increasingly targeted for control through vaccination, and malaria, hitherto the main cause of febrile illness in the region, steadily decreases in some areas.

On January 24, 2009, the International Vaccine Institute (located in Seoul, Republic of Korea) and the Kenya Medical Research Institute (Kilifi, Kenya) cohosted a meeting to review published and unpublished data on invasive disease caused by *Salmonella* Typhi and *S. enterica* non-Typhi serotypes in sub-Saharan Africa. The meeting was supported by the Swedish International Cooperation and Development Agency and held in conjunction with the Seventh International Conference on Typhoid Fever and Other Invasive Salmonelloses in Kilifi, Kenya. Twenty-eight investigators from 14 research sites in sub-Saharan Africa and 5 international institutions presented data on invasive bacterial diseases that focused on salmonelloses and discussed the way forward for defining invasive *Salmonella* in sub-Saharan Africa.

Characteristics of 11 fever surveillance sites were compared (Table). Most sites identified both *Salmonella* Typhi and *S. enterica* non-Typhi serotypes as causes of invasive infections. *Salmonella* Typhi seemed to be affecting older children and young adults, whereas invasive *S. enterica* non-Typhi serotypes were more common in infants, toddlers, and HIV-infected adults. The ratio of *Salmonella* Typhi to invasive *S. enterica* non-Typhi serotypes varied greatly across sites. In infants, the incidence of *S. enterica* non-Typhi serotypes was similar to the incidences of *S. pneumoniae* and Hib in some sites. Predominant *S. enterica* non-Typhi serotypes differed by site: *Salmonella* serotypes Typhimurium and Enteritidis accounted for a large proportion of the *S. enterica* non-Typhi serotypes identified, but *Salmonella* serotypes Dublin, Hvittingfoss, Heidelberg, Isangi, and Stanleyville also were reported as noteworthy causes of infection with *S. enterica* non-Typhi serotypes. Antimicrobial drug resistance patterns of *S. enterica* non-Typhi serotypes were heterogeneous by site; however, the levels were high enough to warrant concern. The lack of rapid diagnostic tests for *S. enterica* non-Typhi serotypes infection was also a concern because poor diagnostics contribute to the inaccurate reporting of disease, and treating febrile illnesses only with antimalarial medication is widely practiced, particularly in malaria-endemic areas. Culture confirmation is considered the accepted standard but is not always performed because it is expensive and requires considerable infrastructure and expertise and, considering its sensitivity, is likely to grossly underestimate the actual prevalence of disease.

**Table:** Characteristics of fever surveillance sites presented during meeting to form a fever surveillance consortium in sub-Saharan Africa, Kilifi, Kenya, January 24, 2009*

Location	Type of surveillance	Age group	Inclusion criteria	Laboratory tests
Agogo, Republic of Ghana (rural)	Hospital-based, passive	>2 m	History of (72 h) or acute fever and severe disease	Blood culture, malaria smear
Kilifi, Kenya (rural)	Hospital-based, passive	Pediatric population	All pediatric admissions	Blood culture, HIV test, malaria smear
Bamako, Mali (urban and rural)	Hospital-based, passive	0–15 y	Fever >39°C, clinical syndrome suggestive of invasive bacterial disease	Blood culture, malaria smear
Lambaréné, Gabon (rural)	Hospital-based, passive	All ages (at physicians request)	Severe disease with fever	Blood culture, HIV test, malaria smear
Muheza, Tanzania (rural)	Hospital-based, passive	2 m–14 y	History of (72 h) or acute fever and severe disease	Blood culture, HIV test, malaria smear
Moshi, Tanzania (urban and rural)	Hospital-based, passive	>2 mo	Child: history (72 h) or acute fever and severe disease; Adult: fever >38.0°C (oral)	Blood culture, HIV test, malaria smear
Nairobi, Kenya (urban)	Population-based, active with household- and clinic-based surveillance	All ages	Fever >38.5°C or suspected severe pneumonia	Blood culture, malaria smear, HIV test, NP/OP swabs of patients meeting pneumonia or influenza criteria
Pemba, Zanzibar (rural)	Hospital-based, passive	>2 mo	Fever >3 d	Blood culture, HIV test, malaria smear
Abuja, Nigeria (urban and rural)	Hospital based, passive	2–59 mo	Fever <14 d	Blood culture, malaria smear, HIV test, APR
Blantyre, Malawi (urban and rural)	Hospital-based, passive	Few neonates; largely >2 mo	Any nonmalarial febrile illness/ suspicion of invasive bacterial infection	Blood culture, CSF, HIV test, malaria smear, radiology, sputum smear and culture
South Africa (countrywide)	Active laboratory surveillance	All ages	NA; all invasive/noninvasive salmonellae isolated	Specimen culture

*NP, nasopharyngeal; OP, oropharyngeal; APR, acute-phase reactant; CSF, cerebrospinal fluid; NA, not available.

Variability in findings across sites can be explained partly by differences in setting (e.g., urban/rural, hospital-based/population-based), criteria for enrollment, age groups included, and laboratory methods. The use of standard protocols across sites would make combining data for analysis and comparing and contrasting results possible. The Diseases of the Most Impoverished Program conducted by the International Vaccine Institute in Asia produced comparative data on typhoid and paratyphoid fever infections through implementation of common epidemiologic and laboratory methods across multiple sites (*11*). An array of evidence was generated on the need to control typhoid fever through the program's multidisciplinary approach, which also included policy, economic, and sociobehavioral studies and vaccine demonstration projects. A similar approach may be emulated in sub-Saharan Africa for the studies on invasive salmonelloses.

Meeting participants concluded that existing epidemiologic information about invasive salmonelloses in sub-Saharan Africa remains fragmentary and hindered by heterogeneous study methods. Participants agreed on an urgent need to collect better data on invasive *Salmonella* infections in these areas. Collection of standardized, multicountry, multidisciplinary data on the prevalence of, risk factors for, and costs of these infections is essential for developing consensus at the regional, national, and international levels in establishing appropriate control measures. The optimal study approach is to use existing fever surveillance sites and to standardize epidemiologic and laboratory procedures across sites. In addition, at sites where the prevalence of disease associated with *Salmonella* Typhi is known and considerable, vaccine demonstration projects should be conducted to control the disease. The framework for a multicenter proposal was discussed, and the group agreed on the creation of an African salmonelloses initiative for research and advocacy. This consortium will undertake much-needed standardized multicountry studies to yield evidence about typhoid and other invasive salmonelloses and the effectiveness of typhoid vaccines in sub-Saharan Africa, which ultimately can lead to deployment of preventive interventions, including vaccination, against invasive *Salmonella* infections.

Investigators participating in the meeting were Katrin Kösters, Albert Schweitzer Hospital, Lambaréné, Gabon; Jürgen May, Bernhard Nocht Institute for Tropical Medicine, Hamburg, Germany; Robert F. Breiman, US Centers for Disease Control and Prevention, Nairobi, Kenya; John A. Crump, Duke University–Kilimanjaro Christian Medical Centre Collaboration, Moshi, Tanzania; John Clemens, Michael Favorov, Florian Marks, and R. Leon Ochiai, International Vaccine Institute, Seoul, Republic of Korea; Kamala Thriemer, International Vaccine Institute, Pemba, Tanzania; Lorenz von Seidlein, International Vaccine Institute, Zanzibar, Tanzania; Sam Kariuki, Kenya Medical Research Institute, Nairobi, Kenya; Kevin Marsh and Susan C. Morpeth, Kenya; Medical Research Institute–Wellcome Research Program, Kilifi, Kenya; Yaw Adu-Sarkodie, Kwame Nkrumah University of Science and Technology School of Medical Sciences, Kumasi, Republic of Ghana; Denise Dekker, London School of Hygiene and Tropical Medicine, Korogwe, Tanzania; Hugh Reyburn, London School of Hygiene and Tropical Medicine, Moshi, Tanzania; Robert Heyderman, Malawi–Liverpool–Wellcome Trust Clinical Research Program, Blantyre, Malawi; Usman Nurudeen Ikumpayi, Medical Research Council Laboratories, Basse, The Gambia; Stephen Obaro, Michigan State University, East Lansing, Michigan, USA; George Mtove, National Institute of Medical Research, Amani, Tanzania; Saidi M Ali and Shaali Ame, Public Health Laboratory–Ivo de Carneiro, Pemba, Tanzania; Gordon Dougan, Sanger Institute, Cambridge, UK; John Wain, Health Protection Agency, London, UK; Karen H. Keddy, National Institute for Communicable Diseases, Johannesburg, Republic of South Africa; Ben Amos, Teule Hospital, Muheza, Tanzania; Myron M. Levine, Center for Vaccine Development, Bamako, Mali; and William Mwengee, World Health Organization, Dar es Salaam, Tanzania.
